# Case Report: Idiopathic calvarial hyperostosis and concurrent nasal discharge in a 22-week-old Staffordshire bull terrier

**DOI:** 10.3389/fvets.2025.1616797

**Published:** 2025-09-11

**Authors:** Victoria Travail, Beatriz Moreno-Aguado, Claudio Motta, Beatriz Garcia, Darren Kelly

**Affiliations:** ^1^Southern Counties Veterinary Specialists, Ringwood, United Kingdom; ^2^Veterinary Pathology Group, Exeter, United Kingdom

**Keywords:** calvarial, hyperostosis, idiopathic, Staffordshire bull terrier, dog

## Abstract

A 22-week-old Staffordshire bull terrier presented with acute onset of bilateral mucopurulent nasal discharge and lethargy. Clinical examination revealed pyrexia and pain upon palpation of the head, accompanied by marked asymmetrical swelling. Computed tomography (CT) of the head showed severe thickening of multiple calvarial bones with periosteal reaction, with small defined areas of fluid accumulation and contrast enhancement of the osteopenic areas. There was mild thickening of the mucosa of the frontal sinuses suggestive of sinusitis, fluid in the left nasal passage, choanae, and nasopharynx. Histological examination of biopsies of the bone revealed periosteal reaction with fibrosis, neutrophilic and histiocytic inflammation, and necrosis. Culture yielded negative results. The dog was diagnosed with idiopathic calvarial hyperostosis and medically managed with methadone, paracetamol, and meloxicam. At recheck 10 days later, all clinical signs had resolved except for mild persistence of skull asymmetry. To the authors’ knowledge, this is the first report of idiopathic calvarial hyperostosis in a Staffordshire bull terrier. The presence of bilateral purulent nasal discharge represents a novel clinical sign not previously reported.

## Introduction

Idiopathic calvarial hyperostosis (CHS) is a non-neoplastic and asymmetrical proliferation of the flat bones of the skull (1–3). It was initially described in Bullmastiffs but has recently been reported in other breeds such as English Springer Spaniels, Bull Terriers, Weinmaraners, Dalmatians and Labrador Retrievers (1–8). It is unclear whether male dogs are predisposed (1–3). The most common clinical signs reported are pain, asymmetrical swelling of the head, lymphadenomegaly, eosinophilia, and fever (1, 4, 9). The disease is typically self-limiting and tends to resolve with skeletal maturity, but analgesic medications are often required during the acute phase (5). To the authors’ knowledge, this condition has not been previously reported in the Staffordshire bull terrier. The presence of bilateral purulent nasal discharge represents a novel clinical sign not previously reported.

## Case presentation

A 22-week-old male Staffordshire bull terrier was presented to the primary care practice for acute onset of bilateral mucopurulent nasal discharge, pyrexia (39.9°C), swelling of the head, hyporexia, and lethargy. Radiographs of the head were performed and revealed thickening of the frontal bone. He was treated with amoxicillin-clavulanic acid (20 mg/kg every 8 h intravenously) and paracetamol (10 mg/kg every 8 h intravenously). Twenty-four hours later, he was referred to a multi-disciplinary referral hospital for further investigation due to lack of improvement in the clinical signs.

On physical examination, the dog was bright, alert, and responsive. The rectal temperature was 39.7°C. He had marked asymmetrical swelling on the left side of the forehead. Palpation of the skull was not possible due to apparent discomfort. The rest of the clinical examination was unremarkable.

The dog was hospitalised and received crystalloid fluid therapy at a maintenance rate of 2.5 mL/kg/h. He was started on methadone (0.2 mg/kg every 2 h intravenously) and paracetamol (15 mg/kg every 8 h intravenously) at the referral hospital, while amoxicillin-clavulanic acid (Co-amoxiclav 500 mg/100 mg powder for solution for injection; Sandoz, United Kingdom; 20 mg/kg intravenously every 8 h) was continued from prior treatment by the primary veterinarian until further investigations were performed.

## Investigations

Complete blood count revealed mild monocytosis (3.25 × 10^9^/L; reference: 0.16–1.12). Serum biochemistry revealed mild hyponatraemia (136 mmol/L; reference: 139–154) and hypochloraemia (97 mmol/L; reference: 99–119).

A CT of the head was performed with the patient in sternal recumbency. The imaging series included unenhanced and post-contrast venous phases. The post-contrast series was acquired following intravenous administration of 2 mL/kg of iodinated non-ionic contrast agent (Omnipaque 300 mg/mL solution for injection, Iohexol, GE Healthcare, Oslo, Norway) using automated injection. Images were displayed using bone window settings (window level 300 HU, window width 1,500 HU) for high-frequency reconstruction algorithm, and soft tissue window (window level 50 HU, window width 350 HU) for medium-frequency reconstruction algorithm. They were evaluated using a commercial DICOM viewer (Osirix, version 7.0.1, Pixmeo, Switzerland).

**Figure 1:**
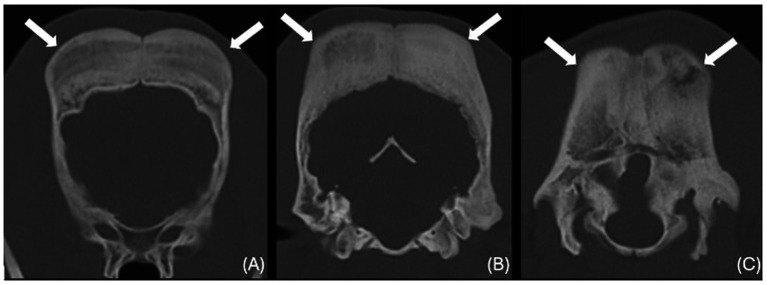
Transverse CT images of the frontal **(A)**, parietal **(B)** and occipital bones **(C)** (window level 300HU, widow width 1500HU) unenhanced, showing severe thickening and sclerosis (white arrows).

**Figure 2:**
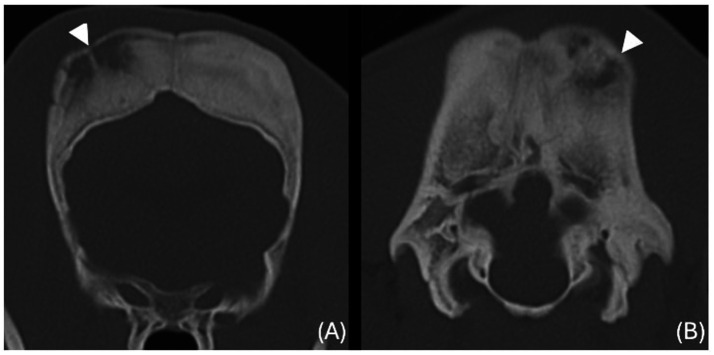
Transverse CT images of the frontal **(A)** and occipital **(B)** bones (window level 300HU, widow width 1500HU) unenhanced, showing ill-defined hypoattenuating regions with punctate cortical lysis (white arrowheads).

**Figure 3:**
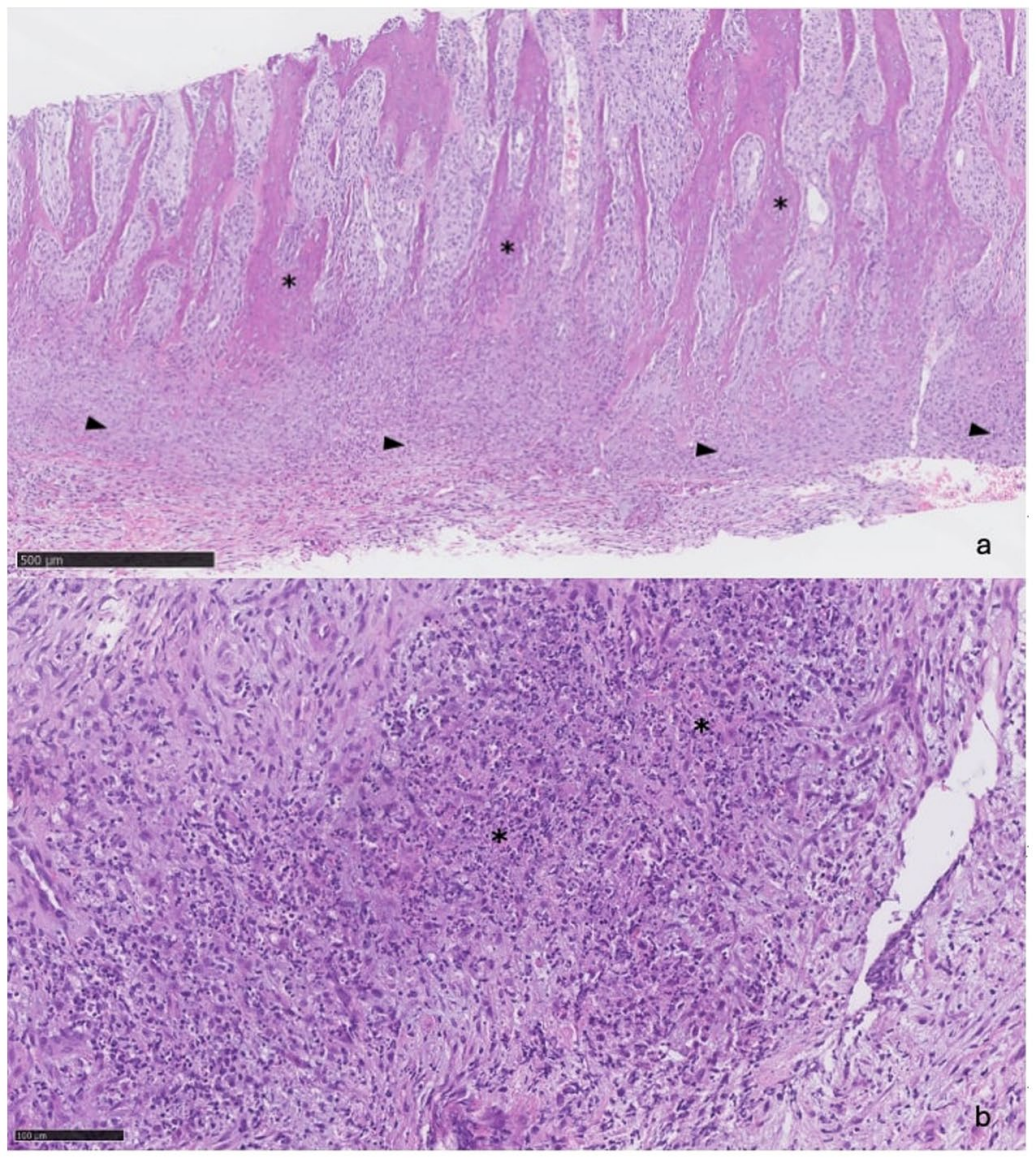
Transverse CT images of the parietal bones unenhanced **(A)** and post contrast venous phase **(B)**, showing the areas of lysis being occupied by fluid to soft tissue attenuating material (black arrows) and ill-defined hypoattenuating, contrast enhancing changes in the right temporal muscle (white arrows).

The images revealed severe, bilateral and symmetrical thickening of the parietal, occipital and frontal bones, as well as the squamous part of the left temporal bone (Figure 1, white arrows). The lesions were characterised by prominent bone sclerosis, which appeared lamellated in some areas, as well as more focal irregular areas of hypoattenuation with punctate lysis of the cortex (at the right frontal, left parietal, and left occipital bones) (Figure 2, white arrowheads). The osteopenic areas were occupied by mixed soft tissue and fluid attenuation with patchy contrast enhancement (Figure 3, black arrows). Irregular margination with smooth periosteal reaction of the intracranial side of the occipital and parietal bones was also noted. The os tentorium was thickened and sclerotic, especially on the left side.

Subtle, ill-defined areas of hypoattenuation were noted at the right temporal muscle overlying the temporal bone with associated swelling (Figure 3, white arrows). These were more conspicuous on the post-contrast series, as ill-defined contrast enhancement was noted surrounding them. Similar changes were noted on the left temporal muscle in the region of the left occipital bone.

**Figure 4:**
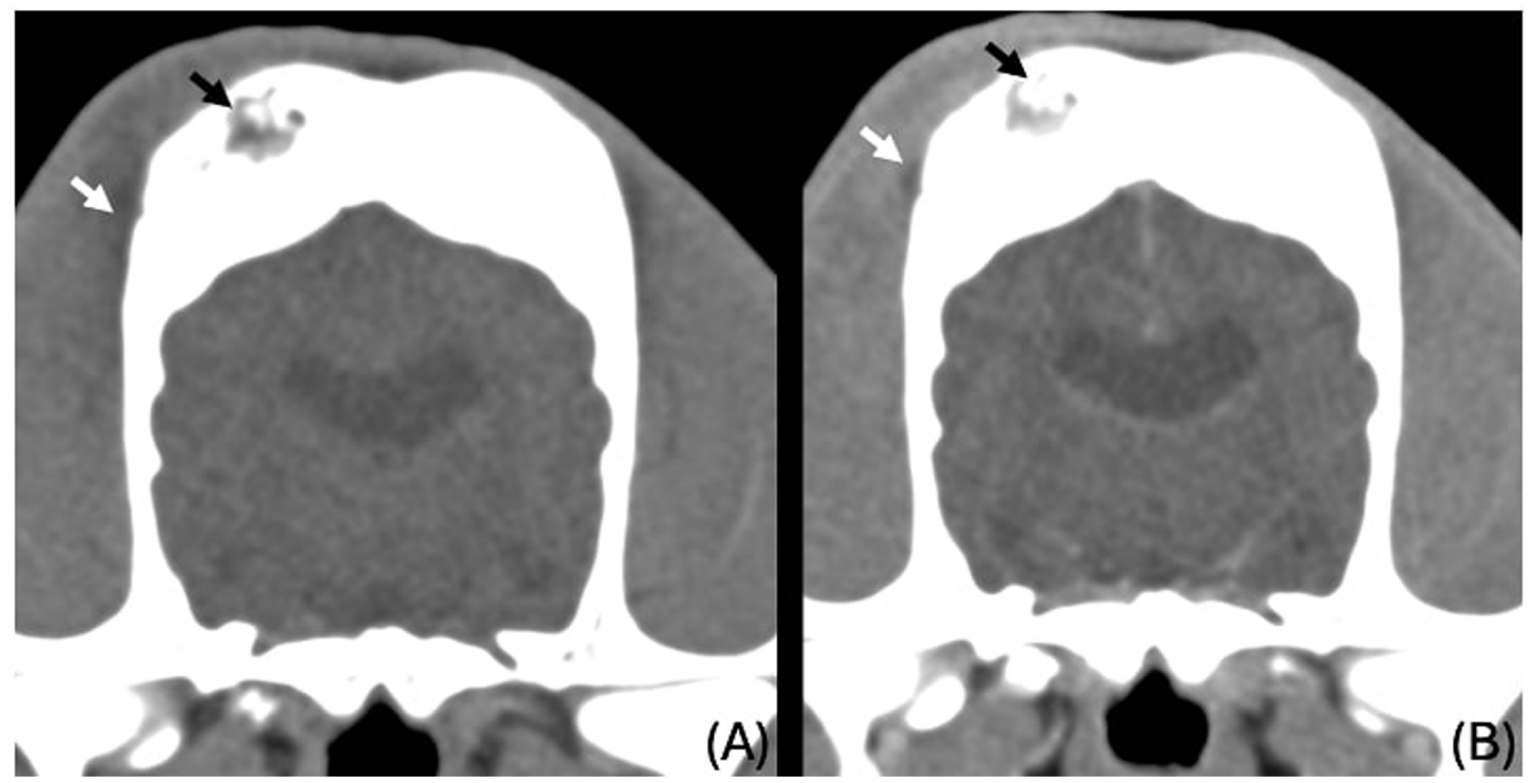
**(A)** (5x): Trabeculae of reactive bone (asterisks) separated by bundles of fibroblasts and expanded periosteum (arrowheads). **(B)** (20x): Foci of necrosis (asterisks).

There was a small volume of fluid-attenuating, non-contrast-enhancing material in the ventral nasal concha and ventral nasal meatus (more marked on the left) with no evidence of turbinate destruction (Figure 4, white arrows and arrowheads). There was also mild fluid accumulation in the left aspect of the choana and left aspect of the nasopharynx. The remaining nasal cavity was within normal limits. There was irregular and multifocal mild mucosal thickening in the dorsal aspect of the frontal sinuses, which also showed mild contrast enhancement.

Mandibular and medial retropharyngeal lymphadenomegaly were reported. The lymphadenopathy was believed likely reactive.

Bone biopsies were obtained from the abnormal frontal bone using a 9-gauge Jamshidi needle. Histopathologic assessment confirmed that the biopsies consisted mainly of anastomosing trabeculae of reactive bone. Present between the trabeculae were bundles of plump fibroblasts that merged with a band of dense fibrous tissue expanding the periosteum (Figure 4A). Intermingled with the fibroblasts were small clusters of neutrophils and some macrophages. In one of the sections, the fibrous tissue exhibited multifocal areas of necrosis, characterised by extensive accumulations of eosinophilic granular debris and nuclear fragments (Figure 4B). Some extravasated erythrocytes were also seen.

Bacterial (aerobic and anaerobic) and fungal cultures were negative. Based on clinical signs, imaging findings, lesion location, and histological features (including mild neutrophilic inflammation and areas of necrosis), the dog was diagnosed with idiopathic calvarial hyperostosis.

## Treatment

The dog was hospitalised and treated with methadone (Comfortan 10 mg/mL solution for injection; Dechra, United Kingdom; 0.2–0.3 mg/kg intravenously every 4 h), paracetamol (Paracetamol 10 mg/mL solution for infusion; Braun, Germany; 15 mg/kg intravenously every 8 h). Following investigations, the amoxicillin-clavulanic acid was discontinued. After 48 h, the dog’s demeanour and appetite improved. His temperature normalised, and since he was more comfortable, the opioids were discontinued. He was discharged with paracetamol (Paracetamol 200 mg; BOVA, United Kingdom; 10 mg/kg PO every 12 h) and meloxicam (Loxicom oral suspension; Norbrook; 0.1 mg/kg every 24 h orally).

## Outcome and follow-up

The owner reported an immediate and significant improvement in the dog’s demeanour. Ten days following discharge, the swelling of the forehead had visibly reduced, and since the dog seemed comfortable, analgesic medications were discontinued.

## Discussion

Idiopathic calvarial hyperostosis has been reported mostly in Bullmastiffs (1–8). In this case report, the dog presented with asymmetrical swelling and discomfort on palpation of the head, fever, and lymphadenopathy, which are compatible signs of CHS (1, 4, 9). The disease was confirmed by diagnostic imaging and histological evaluation of the lesions. This is the first report of CHS in a Staffordshire bull terrier dog.

Other signs reported with the condition include hydrocephaly, seizures, lameness, exophthalmos, and purulent osteomyelitis (1, 3, 4, 9). The dog described in this case also presented with bilateral mucopurulent nasal discharge. The CT scan revealed changes compatible with sinusitis and rhinitis. These changes could be due to the progression of the inflammatory process affecting the calvarial bones into the mucosa of the frontal sinuses. Although histopathological evaluation of the nasal mucosa and cytological evaluation of the nasal discharge were not performed, and therefore definitive conclusions cannot be drawn, the temporal association of the clinical signs raises suspicion that the same underlying inflammatory process may be responsible for both the calvarial hyperosteosis and the rhinitis/sinusitis. To the authors’ knowledge, nasal discharge has never been reported in association with CHS before, and it could represent a previously unrecognized feature of the disease.

Similarities between CHS and human infantile cortical hyperostosis (HICH) have been noted. Subperiosteal new bone formation developing in long bones and the mandible of newborns is characteristic of HICH (9–11). As in CHS, patients suffering from HICH present with pain and fever, and the disease regresses within a few months (10, 11). However, the distribution of the disorder differs; HICH, as opposed to CHS, is symmetrical and bilateral (10, 11). In both diseases, thickening of the cortical bone can be seen on radiography (10, 11). In humans, an anomaly of collagen secondary to a mutation of the **COL1A1** gene has been suspected (9). Prostaglandins E1 and E2 have also been suggested to be involved in the pathogenesis, with increased serum concentrations in some affected children (6).

Previous reports of CHS responsive to antibiotics have raised suspicion of a bacterial agent being responsible for the onset of the disease in some cases (1, 2). Osteomyelitis was considered in this case, but no infectious agent was identified on histological examination or culture. The patient had already been treated with antibiotics before biopsies were collected, which could have affected the results. However, despite being on antibiotics, the dog was still pyrexic on presentation. Furthermore, trauma was not reported in the clinical history, and no compatible clinical signs or imaging changes such as bruises, wounds, or fractures were noted. A traumatic event was therefore considered unlikely. As the disease has been reported in various breeds to date, a consistent genetic predisposition appears less likely. Additionally in this case, none of the littermates were reported to be affected by similar clinical signs, supporting the sporadic nature of the disease in this case, although a hereditary component cannot be entirely excluded without further genetic evaluation.

Another differential considered was canine craniomandibular osteopathy (CMO), which, similarly to CHS, occurs in young dogs and is self-limiting (1, 2, 4–6). CMO predominantly affects the mandibular bone and joints; the tympanic bullae, and occasionally the occipital and parietal bones can also be involved. In contrast to CHS, CMO lesions are bilateral and symmetrical (1, 3, 12–14). Dogs with CHS or CMO present with pain and pyrexia. Due to the localisation of CMO lesions in the mandible, affected dogs may show discomfort when opening the mouth, anorexia, and ptyalism (14, 15). Histologically, CMO differs by infiltration of connective tissue and inflammatory cells within the medullary cavities of the affected bone (1, 12, 14). In CHS, the medullary bone is unremarkable, with histological changes limited to the subperiosteal area, including fibrovascular infiltration between the trabeculae and inflammatory cells (1, 3, 12, 13).

Changes consistent with calvarial osteolysis have been described in 25% of cases of CMO (16), and this case report showed compatible bone lysis changes identified on CT. This is likely due to the superior resolution of CT compared to radiographs and the lack of superimposition, allowing clearer identification of mineralisation loss. In this dog, multifocal necrosis was identified histologically. To the authors’ knowledge, this is the first case report to describe this feature in a dog diagnosed with idiopathic calvarial hyperostosis. This could represent a novel finding or may reflect the fact that bone biopsies are not always performed, with many CHS diagnoses relying solely on radiographic findings. Furthermore, if the necrosis is localised, biopsies may miss it if samples are taken from unaffected areas. The necrosis seen in this case could be attributed to compromised blood supply due to vessel compression, thrombus formation, or as a consequence of prolonged inflammation.

No treatment to prevent progression of the lesions has been reported. Dogs with CHS are managed palliatively with anti-inflammatories and analgesics to reduce discomfort during the active phase. It is believed to be self-limiting, with lesion progression halted or even regressing as the patient matures (1–7). In our case, clinical signs had significantly improved within 10 days of treatment initiation. However, we acknowledge that a 10-day follow-up period is relatively short to definitively confirm complete remission. While the improvement was encouraging, longer-term follow-up would be required to confirm sustained remission. Unfortunately, no further clinical assessments were available due to owner constraints. Additionally, no repeat imaging was performed during the follow-up period, which we have noted as a limitation. Although clinical signs (e.g., facial swelling, and nasal discharge) had improved, we cannot confirm radiological resolution without follow-up imaging.

## Conclusion

Idiopathic calvarial hyperostosis should be considered in Staffordshire bull terriers with compatible clinical signs and imaging findings. Mucopurulent discharge could also be a concurrent clinical sign in dogs suffering from CHS. The disease affects young dogs and is self-limiting as they grow. For this reason, they do not require specific treatment, except for pain management during the onset of the disease.

## Data Availability

The original contributions presented in the study are included in the article/Supplementary material, further inquiries can be directed to the corresponding author/s.

## Supplementary material

Supplementary Figure 1 Transverse CT images of the frontal **(A)**, parietal **(B)** and occipital bones **(C)** (window level 300HU, widow width 1500HU) unenhanced, showing severe thickening and sclerosis (white arrows).
