# Supplementary figures and images for: Case Report: Idiopathic calvarial hyperostosis and concurrent nasal discharge in a 22-week-old Staffordshire bull terrier
Source: Front Vet Sci. 2025 Sep 11;12:1616797. doi: 10.3389/fvets.2025.1616797 (PMC12461860; doi:10.3389/fvets.2025.1616797)

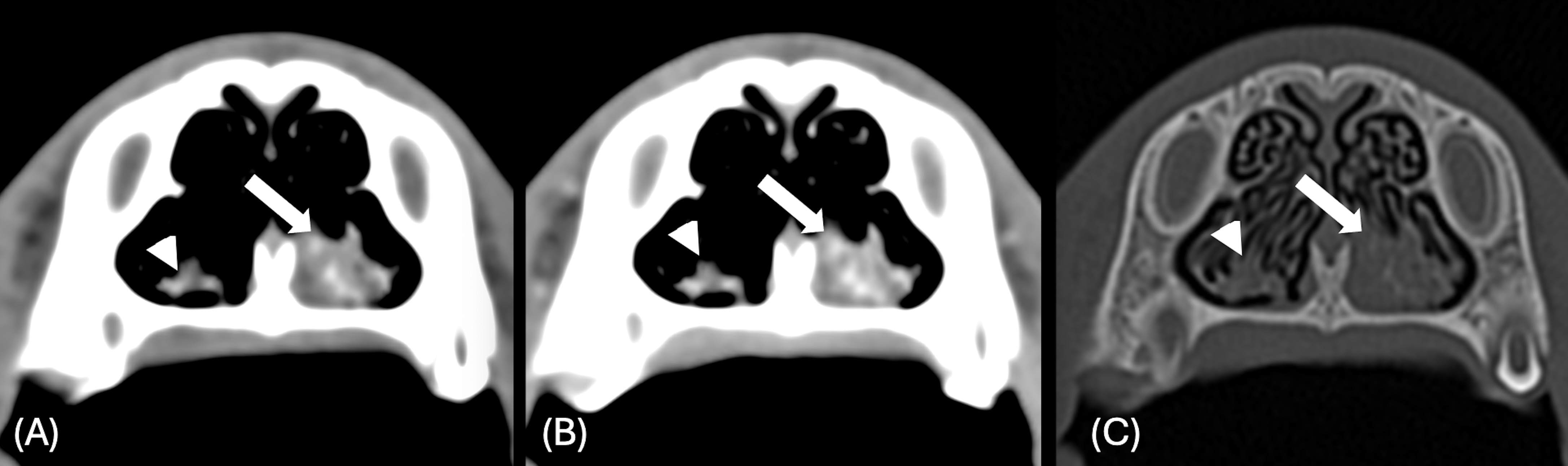

Supplement: Supplementary Figure 1 — Transverse CT images of the frontal (A), parietal (B) and occipital bones (C) (window level 300HU, widow width 1500HU) unenhanced, showing severe thickening and sclerosis (white arrows). [file Image_1.jpeg]
